# Hypoxemia Surrounding Enteral Feeds in Preterm Infants Receiving Extended Continuous Positive Airway Pressure

**DOI:** 10.21203/rs.3.rs-10755917/v1

**Published:** 2026-09-20

**Authors:** Rachna Mamidi, Kelvin MacDonald, Sheila Markwardt, Alicia Johnson, Mitzi Donabel Go, Matthew Olson, Kristin Milner, Robert Schelonka, Cindy McEvoy

**Affiliations:** University of Missouri; Oregon Health & Science University; Oregon Health & Science University; Oregon Health & Science University; Oregon Health & Science University

## Abstract

**Objectives:**

Hypoxemia is common in preterm infants and associated with morbidities. We evaluated the relationship between OG feeds and hypoxemia and the effect of extending CPAP.

**Design/Methods:**

In this prospective substudy of the NHLBI-supported trial (NCT04295564), stable preterm infants were randomized to two additional weeks of CPAP (eCPAP) or CPAP discontinuation (dCPAP) to room air. Oxygen saturation was analyzed before, during, and after feeds in 30 infants. The primary outcome was percent time with SpO_2_<90%; secondary outcomes included percent time with SpO_2_<80%, IH episodes < 90% and < 80%.

**Results:**

Infants randomized to eCPAP spent significantly less time with SpO_2_<90% than dCPAP infants before, during, and after feedings, including a 6.8 percentage-point reduction post-feed (p = 0.006). No differences were observed for SpO_2_<80%. eCPAP infants also experienced fewer IH episodes < 90% over the two-week study period (p = 0.02)

**Conclusions:**

Extending CPAP reduced hypoxemia in preterm infants. OG feedings were not associated with increased hypoxemia or IH.

## INTRODUCTION

Intermittent hypoxemia (IH) is common in preterm infants and is associated with adverse neonatal outcomes, including bronchopulmonary dysplasia and retinopathy of prematurity.^1–3^ Enteral feeding is frequently suspected to precipitate apnea, bradycardia, and oxygen desaturation, often leading clinicians to interrupt or delay gavage feedings.^4,5^ Proposed mechanisms include gastric distention, gastroesophageal reflux (GER), and reduced functional residual capacity (FRC) resulting from the highly compliant chest wall of preterm infants. However, evidence linking enteral feeding with cardiorespiratory instability remains limited and inconclusive,^6,7^ and prior studies have primarily evaluated more severe hypoxemia despite evidence that relatively “mild” decreases in pulse oximeter oxygen saturation (SpO_2_) to < 90% are associated with increased respiratory morbidity and mortality.^8^

Nasal continuous positive airway pressure (CPAP) improves oxygenation by maintaining FRC and upper airway patency.^9–12^ In a recent secondary analysis of the NHLBI-supported eCPAP trial (NCT04295564), we demonstrated extending CPAP reduced overall intermittent hypoxemia and improved oxygenation during a two-week treatment period.^13^ Whether these benefits extend to the physiologic challenges surrounding enteral feedings remains unknown. Therefore, we performed a physiologic substudy of the eCPAP randomized controlled trial (RCT) to determine whether enteral feeding is associated with hypoxemia in stable preterm infants and whether extending CPAP modifies oxygenation before, during, and after feedings. We hypothesized that infants randomized to extended CPAP would spend less time in hypoxemia and experience fewer IH episodes during and after feeds than infants randomized to CPAP discontinuation.

## METHODS

### Study Design

This was a single-center prospective physiologic substudy of the NHLBI supported trial “Extending CPAP Therapy in Stable Preterm Infants to Increase Lung Growth and Function: A Randomized Controlled Trial (eCPAP Trial; NCT04295564)”, conducted in the level IV Neonatal Intensive Care Unit (NICU) at Oregon Health & Science University (OHSU). This substudy evaluated oxygenation surrounding enteral feedings in stable preterm infants randomized to extended CPAP or CPAP discontinuation. The study was approved by the OHSU Institutional Review Board. Informed consent was obtained for each participant, and all procedures were conducted in accordance with the ethical principles of the Declaration of Helsinki.

In the parent RCT, preterm infants who met prespecified respiratory stability criteria were randomized to two weeks of extended CPAP (eCPAP) in room air versus CPAP discontinuation (dCPAP) to unsupported room air. The RCT demonstrated that eCPAP significantly improved alveolar volume, lung diffusion capacity, and forced expiratory flows after discharge from the NICU, at about 6 months of corrected age.^14^ A subsequent secondary analysis of the RCT demonstrated eCPAP also significantly reduced frequency and duration of intermittent hypoxemia while increasing mean oxygen saturation during the two-week treatment period.^13^

### Participants

This study included preterm infants born > 24 and ≤ 32 weeks gestation who required CPAP ≥ 24 hours for clinical care and met specific respiratory stability criteria for ≥ 12 hours at < 35 weeks postmenstrual age .^13,15,16^ Infants were randomized to two weeks of eCPAP or dCPAP, which is the standard of care at the OHSU NICU. Of the 100 patients enrolled in the RCT, the final 30 were prospectively enrolled in this preplanned physiologic study. Eligible infants were receiving intermittent bolus enteral feedings, had continuous pulse oximetry data available for > 75% of the 14-day treatment period, and were singleton births. The sample size was determined pragmatically by the number of infants enrolled during the substudy period. A CONSORT flow diagram of participant enrollment into the eCPAP RCT and nested physiologic substudy is shown in Supplement.

### Data Collection

Pulse oximeter oxygen saturation was collected continuously during the two weeks of randomized treatment with a second pulse oximeter (Radical 7; Masimo Corp., Irvine, CA, USA) separate from patient care, which had its alarms turned off and hidden from the bedside caretaker. This pulse oximeter has a 2-second sample rate and an onboard Masimo^®^ Trace^™^ software filter that continually analyzes plethysmography data to validate measurements, which allows granular quantification of IH episodes. By comparison, conventional bedside pulse oximeter settings use longer averaging times with an 8-second window to decrease alarm fatigue for the bedside caretakers.

Feed start and stop times for all enteral feedings during the 14-day study period were obtained from the electronic medical record. All enteral feedings occurring during the 14-day randomized treatment period for each infant were included in the analysis, including gavage feedings administered by gravity (approximately 20 minutes) or enteral infusion pump (30–60 minutes) and oral feedings. For each feeding, physiologic data were analyzed during the 60-minute period preceding feeding (pre-feed), throughout the entire feeding interval, and during the 60-minute period following completion of feeding (post-feed). Feedings were scheduled such that these analysis intervals did not overlap. A study investigator manually reviewed the pulse oximetry data for each interval. Brief periods of poor signal quality or motion artifact were removed while the remainder of the interval was retained. Intervals were included in the analysis if ≥ 75% of the pulse oximetry data were valid.

### Statistical Analysis and Outcomes

Patient and maternal characteristics were summarized using mean and standard deviation (SD) or median and interquartile range (IQR) for continuous variables and number and percentage for categorical variables. Physiologic measurements were summarized using estimates from unconditional regression models with clustered robust standard errors to account for repeated measurements within patients. Results are presented as estimated means or counts with 95% confidence intervals.

Analyses were conducted by a statistician blinded to treatment arms on the following comparisons of interest: 1) the pre-feed interval versus the combined during and after interval in the dCPAP group, 2) the pre-feed interval versus the combined during and after interval in the eCPAP group, 3) the dCPAP versus eCPAP groups during the pre-feed period, 4) the two groups during the feed period, and 5) the two groups during the post-feed period. The primary outcome was percentage of time with oxygen saturation < 90%, as this threshold has been shown to be associated with unfavorable respiratory outcomes in preterm infants.^8^ Secondary outcomes included percentage of time with oxygen saturation < 80%, number of IH episodes with SpO_2_ <90% (for 10-300s), and number of IH episodes with SpO_2_ <80% (for 10-300s), using previously published IH duration criteria.^8^ Percentage of time in hypoxemia analyses were conducted using fractional regression, as this method restricts the outcome from 0 to 1, appropriate for percentage scales. Number of IH episode analyses were conducted using negative binomial models which are suitable for count data and included an offset variable to account for varying number of minutes observed per patient. All models controlled for sex, gestational age, and age at randomization, and standard errors were clustered by patient to account for repeated measurements.

To assess model fit, we examined model residuals to see how well the predicted and observed values lined up. Jackknife standard errors were calculated and compared with the original analyses.

Data management was conducted using SAS software (Version 9.4, SAS Inc., Cary, NC), and analyses were run using Stata software (Version 16.1, StataCorp, College Station, TX).

## RESULTS

The last thirty infants were included in the physiologic substudy, with 15 randomized to eCPAP and 15 to dCPAP (Supplement). Baseline maternal and infant characteristics were generally similar between groups (Table 1).

Model-estimated physiologic measurements per feeding episode, including the pre-, during, and post-feed intervals are shown in Table 2. Mean oxygen saturation remained stable across feeding intervals in both groups. Infants randomized to eCPAP consistently demonstrated higher oxygen saturation, spent less time with oxygen saturation < 90%, and experienced fewer intermittent hypoxemia (IH) episodes than infants randomized to dCPAP across the pre-feed, during-feed, and post-feed intervals.

**Figure 1:**
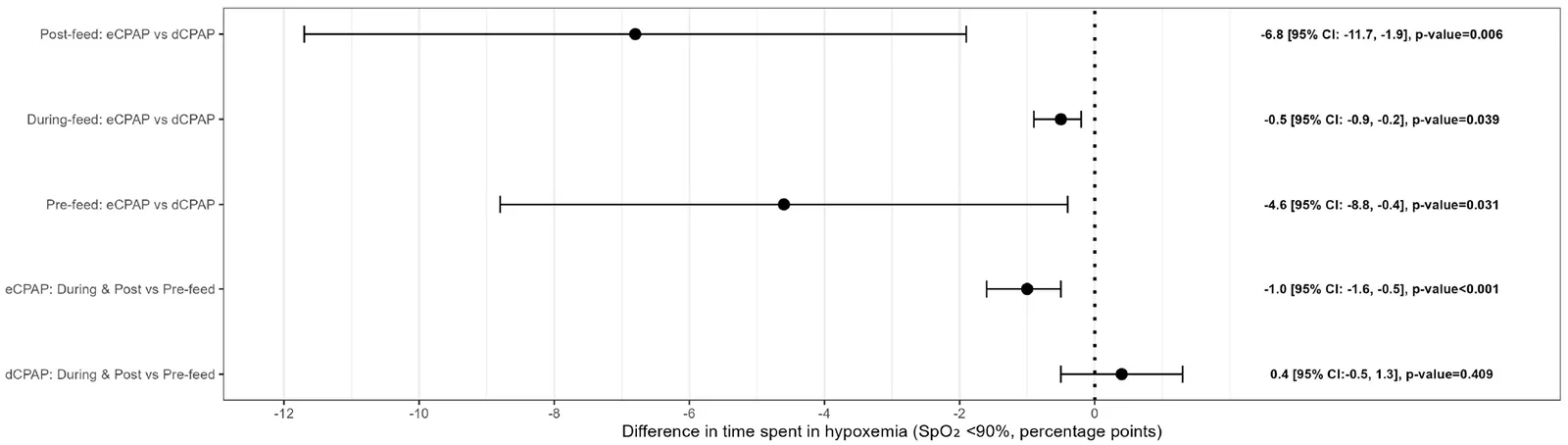
Primary Analysis of Percent of Time SpO_2_ <90% Adjusted percentage point differences in the percentage of time spent with SpO_2_ <90% per feeding episode, comparing feeding intervals within and between respiratory support groups. Shown as point estimate [95% CI]. Estimates were obtained from fractional regression adjusted for sex, age at randomization, and gestational age.

Figure 1 displays the estimated differences in the percentage of time spent in hypoxemia (SpO_2_ <90%) per feeding episode across feeding intervals and respiratory support groups. Within the dCPAP group, there was no significant change in hypoxemia during and after feeding compared with the pre-feed period (difference: 0.4 percentage points; 95% CI: −0.5 to 1.3; p = 0.409). In contrast, infants in the eCPAP group spent significantly less time in hypoxemia during and after feeding relative to the pre-feed period (difference: −1.0 percentage points; 95% CI: −1.6 to − 0.5; p < 0.001). Compared with the dCPAP group, infants receiving eCPAP spent significantly less time in hypoxemia during the pre-feed period (difference: −4.6 percentage points; 95% CI: −8.8 to − 0.4; p = 0.031), during feeding (difference: −0.5 percentage points; 95% CI: −0.9 to − 0.2; p = 0.039), and after feeding (difference: −6.8 percentage points; 95% CI: −11.7 to − 1.9; p = 0.006). Negative values indicate less time spent in hypoxemia in the eCPAP group relative to the comparison group or interval.

Using the 80% oxygen saturation threshold (Table 3), time spent in hypoxemia per feeding episode did not differ significantly between groups across feeding intervals. Relative to the pre-feed period, infants in the dCPAP group spent 0.009 percentage points more time in hypoxemia during and after the feed (95% CI: −0.2 to 0.3; p = 0.942). During the feed, patients in the eCPAP group spent 0.4 percentage points less time in hypoxemia (95% CI: −1.3 to 0.6; p = 0.456) compared with the dCPAP group, and they spent 0.8 percentage points less time in hypoxemia after the feed (95% CI: −1.6 to 0.0; p = 0.060). For total count of IH episodes < 90% around feeds during the entire two-week randomization period, the eCPAP group had 1079.9 less IH episodes than the dCPAP group (95% CI: −1988.4, −171.4; p = 0.02). For total count of IH episodes < 80%, the eCPAP group had 101.9 less IH episodes than the dCPAP group (95% CI: −247.5, 43.8; p = 0.170) (Table 4).

Model diagnostics showed that the models had very small overestimations for most observations, and feeds with more observed hypoxemia tended to be underestimated. This is because the model estimations were fairly moderate, as that was in line with most of the data. The jackknife standard errors were similar to the original values, indicating there were no outliers strongly influencing the models.

## DISCUSSION

We did not find a relationship between oro/nasogastric (OG) feeds and time spent in hypoxemia or IH episodes in preterm infants breathing unsupported room air. This finding contrasts with a widely held belief that cardiorespiratory events increase after OG feeding. Events like apnea, bradycardia and IH often lead to pausing enteral feeds, especially when there is the clinical observation that events occur around feeding. However, cardiorespiratory events are non-specific and as this study suggests, may not be indicative of feeding intolerance but related to other prematurity-related conditions. It has been shown that enteral feeding in infants does cause physiologic changes including a transient rise in oxygen consumption,^17^ decrease in oxygenation saturation,^18,19^ increase in heart rate,^17^ decrease in FRC,^20^ and decrease in lung volume.^21^ Despite these physiologic effects of gavage feeding, our study did not find clinically important IH events around feedings and calls into question the clinical utility of withholding gavage feedings to improve cardiorespiratory stability.

Our study demonstrates that preterm infants randomized to eCPAP spent significantly less time in hypoxemia < 90% across all time intervals – pre-feed, during feed, and post-feed – compared with infants randomized to dCPAP. Although reductions in hypoxemia were observed across all feeding intervals, the largest absolute difference between groups occurred during the post-feed period. Interestingly, infants receiving eCPAP spent less time in hypoxemia during and after feeding than during the pre-feeding interval, whereas no such differences were observed in the dCPAP group. These findings suggest that continued CPAP support enhanced oxygenation stability throughout the feeding period, with the greatest benefit observed after feeding, likely by preserving FRC and upper airway patency.

Nasal CPAP has been shown to reduce nocturnal GER in adult patients with and without obstructive sleep apnea.^22,23^ Many mechanisms have been demonstrated to contribute to the decrease in frequency and duration of GER, including elevation of intraesophageal pressure,^23^ increase in resting lower esophageal sphincter pressure (either through reflex lower esophageal sphincter constriction ^23,24^ or direct mechanical compression of the esophagus by CPAP^25^), and decreased duration of lower esophageal sphincter relaxation.^24^ Despite the evidence in adult humans, there are no studies conducted in preterm infants to investigate this mechanism. In a model of newborn lambs, nasal CPAP inhibited GER and decreased the magnitude and duration of lower esophageal sphincter relaxation.^26^ Further clinical studies are needed to confirm this effect in human preterm infants.

We recently published pulse oximetry outcomes from the entire cohort of infants (n = 95) in the eCPAP RCT who underwent continuous pulse oximetry monitoring for 14 days following randomization.^13^ Infants randomized to eCAP had a significantly higher mean SpO_2_ despite no supplemental oxygen and demonstrated significantly fewer IH episodes across all oxygen saturation thresholds and event durations compared to the dCPAP group. Specifically, IH episodes with SpO_2_ <90% for 10–300 seconds occurred at an estimated mean of 57.6 (SE 23.1) vs 151.7 (SE 23.6) episodes per 24 hour period in the eCPAP and dCPAP groups respectively (adjusted difference 95% CI [−132.2, – 55.9] P < 0.0001). The eCPAP group also demonstrated significantly increased FRC which was inversely correlated with IH burden suggesting that improved lung volume is an important physiologic mechanism underlying the reduction in hypoxemic events.^13^

Our findings build upon previous studies demonstrating that IH is common in extremely low birth weight infants (24–28 weeks’ gestation), with the frequency increasing during the first four weeks of life followed by a plateau and then a slow decline at 6 to 8 weeks.^27^ The etiology of these episodes is multifactorial and incompletely characterized, but important contributors include immature respiratory control resulting in apnea/respiratory pauses, unstable lung volumes/FRC, and a tendency for upper airway collapse. CPAP has been shown to stent the upper airways open,^28^ improve oxygenation,^29^ and promote lung function.^30^ Our study extends these observations by demonstrating, in a RCT, that continuing CPAP for an additional two weeks after infants meet conventional stability criteria results not only in improved lung volumes but also in a marked reduction in IH episodes.^13,16^ Common clinical practice is to wean clinically stable preterm infants off CPAP as soon as possible to minimize potential adverse effects such as air leak, nasal septal breakdown, feeding intolerance, delay of oral feeds, delay of discharge, and impaired infant bonding.^31^ However the eCPAP RCT had no cases of treatment nonadherence in the eCPAP group due to aforementioned adverse effects and both groups achieved full oral feeds and hospital discharge at similar postmenstrual age.

### Strengths and Limitations

A major strength of our study is clear comparison of cohorts as our participants originated from an RCT. Additionally, the OHSU NICU has a clinical consensus guideline for initiation and advancement of enteral feeds in preterm infants. Furthermore, our data collection spanned the entire two-week randomization period which included over 3200 feeds. Almost all the infants in both groups were on full goal feeds at the time of randomization.

There are several limitations to our study. This was an observational study and thus subject to bias and confounding. This physiologic substudy only included the last 30 infants, which may have introduced additional bias, reduced generalizability, and increased risk of overestimating effect sizes. However, our statistical analysis did not find any strong outliers influencing the models. Our study did not include preterm infants with severe respiratory disease, because they did not meet the predefined respiratory stability criteria to be randomized in the parent trial at < 35 weeks of postmenstrual age, therefore our results are not applicable to more critically ill neonates.

## CONCLUSION

In this physiologic substudy of clinically stable preterm infants in the NICU, enteral feeding was not associated with clinically important increases in hypoxemia or intermittent hypoxemia episodes. Infants receiving extended CPAP experienced less hypoxemia and fewer intermittent hypoxemia episodes, before, during, and after feedings than infants breathing unsupported room air, with the greatest benefit observed during the post-feed period. These findings suggest that extending CPAP may improve oxygenation stability throughout the feeding cycle while supporting the safety of enteral feeds in clinically stable preterm infants. Further studies are needed to determine whether improved oxygenation stability surrounding feedings translates into meaningful clinical benefits.

## Supplementary Material

This is a list of supplementary files associated with this preprint. Click to download.

• ConsortOGIHeCPAP081726.docx

## Figures and Tables

**Table 1:** Infant and Maternal Characteristics

	Extended CPAP (N = 15)	Discontinued CPAP (N = 15)
Sex - N (%)		
Male	9 (60.0)	5 (33.3)
Female	6 (40.0)	10 (66.7)
Race - N (%)		
American Indian/Alaskan Native	1 (6.7)	0 (0.0)
Asian	2 (13.3)	1 (6.7)
Black	0 (0.0)	2 (13.3)
White	12 (80.0)	11 (73.3)
More than one race	0 (0.0)	1 (6.7)
Maternal age (yrs) - Mean (SD)	31.7 (5.13)	33.2 (4.89)
Gravida - Median (Q1, Q3)	2 (1, 3)	2 (1, 4)
Antenatal steroids - N (%)	12 (80.0)	12 (80.0)
Cesarean section - N (%)	11 (73.3)	8 (53.3)
Gestational age (wks) - Mean (SD)	30.6 (1.57)	29.8 (1.56)
<28 weeks gestation - N (%)	1 (6.7)	2 (13.3)
Birthweight (kg) - Mean (SD)	1.4 (0.27)	1.3 (0.33)
1-minute APGAR - Median (Q1, Q3)	6 (1, 8)	4 (1, 5)
5-minute APGAR - Median (Q1, Q3)	8 (6, 9)	8 (7, 8)
Surfactant use - N (%)	4 (26.7)	4 (26.7)
Mechanical ventilation - N (%)	5 (33.3)	4 (26.7)
Days of CPAP use before randomization - Median (Q1, Q3)	7.3 (4.44, 19.47)	10.2 (5.75, 22.96)
PMA at randomization (wks) - Mean (SD)	32.5 (0.64)	32.1 (0.56)
Postnatal age at randomization (days) - Median (Q1, Q3)	9 (6, 21)	10 (6, 24)
Weight at randomization (kg) - Mean (SD)	1.5 (0.19)	1.5 (0.27)
Full feeds at randomization - N (%)	13 (86.7)	14 (93.3)
PMA at discharge (wks) - Mean (SD)	38.9 (2.85)	37.9 (3.16)
Weight at discharge (kg) - Mean (SD)	3.0 (0.66)	3.1 (0.61)

**Table 2:** Outcomes by Pre-, During-, and Post-Feeding Intervals and CPAP Group per Feeding Episode

	Estimated Mean Value (95% CI)	
	eCPAP	dCPAP
Average oxygen saturation^1^		
Pre-feed	95.0 [94.1, 95.9]	94.1 [93.1, 95.2]
During feed	95.1 [94.2, 96.1]	94.3 [93.2, 95.4]
Post-feed	95.6 [94.8, 96.4]	94.2 [93.0, 95.3]
Average heart rate^1^		
Pre-feed	166.6 [163.1, 170.1]	161.6 [157.5, 165.7]
During feed	167.4 [166.1, 171.6]	163.4 [159.6, 167.1]
Post-feed	165.5 [162.0, 169.1]	161.5 [157.7, 165.4]
% of time <90% oxygen saturation^2^		
Pre-feed	7.3 [4.9, 9.8]	11.3 [7.3, 15.2]
During feed	8.3 [5.5, 11.1]	11.6 [7.7, 15.4]
Post-feed	5.6 [3.5, 7.6]	11.5 [6.8, 16.3]
% of time <80% oxygen saturation^2^		
Pre-feed	1.3 [0.8, 1.9]	1.9 [1.1, 2.7]
During feed	1.6 [1.1, 2.1]	2.0 [1.2, 2.7]
Post-feed	1.0 [0.7, 1.4]	1.9 [1.1, 2.7]
# of IH episodes (10-300s) <90% oxygen saturation^3^		
Pre-feed	4.5 [2.6, 6.3]	7.7 [4.4, 11.1]
During feed	1.8 [1.2, 2.4]	2.8 [1.7, 3.9]
Post-feed	3.3 [1.8, 4.8]	7.9 [4.1, 11.7]
# of IH episodes (10-300s) <80% oxygen saturation^3^		
Pre-feed	0.6 [0.4, 0.9]	1.1 [0.5, 1.6]
During feed	0.3 [0.2, 0.4]	0.4 [0.2, 0.5]
Post-feed	0.5 [0.3, 0.7]	1.0 [0.4, 1.7]

Values listed as expected means [95% CI]

1 Estimates from unconditional linear regression with clustered-robust standard errors

2 Estimates from unconditional fractional regression with clustered-robust standard errors

3 Estimates from unconditional negative binomial regression with clustered-robust standard errors

**Table 3:** Secondary Analysis of Percent of Time <80% SpO_2_

	Difference of % time in hypoxia [95% CI]^1^	p-value
Combined during + post feed vs pre-feed in dCPAP group	0.009 [−0.2, 0.3]	0.942
Combined during + post feed vs pre-feed in eCPAP group	−0.2 [−0.4, 0.1]	0.168
Pre-feed in eCPAP vs dCPAP group	−0.5 [−1.5, 0.5]	0.338
During feed in eCPAP vs dCPAP group	−0.4 [−1.3, 0.6]	0.456
Post feed in eCPAP vs dCPAP group	−0.8 [−1.6, 0.0]	0.060

1 Estimates from fractional regression with clustered-robust standard errors, covariates include: sex, age at randomization, and gestational age

**Table 4:** Secondary Analysis of Total Number of IH Episodes

	Total Episodes (10-300s) <90% SpO_2_			Total Episodes (10-300s) <80% SpO_2_		
	Estimated Count (95% CI)^1^	Difference (95% CI)^2^	p-value	Estimated Count (95% CI)^1^	Difference (95% CI)^2^	p-value
dCPAP Group	1966.2 [1123.4, 2809.0]	−1079.9 [−1988.4, −171.4]	0.020	261.7 [125.0, 398.4]	−101.9 [−247.5, 43.8]	0.170
eCPAP Group	1026.6 [611.7, 1441.5]			157.2 [95.3, 219.1]		

1 Estimates from unconditional negative binomial regression with clustered-robust standard errors

2 Estimates from negative binomial regression models with clustered-robust standard errors, covariates include: sex, age at randomization, and gestational age

## ABBREVIATIONS

IH: intermittent hypoxemia
GER: gastroesophageal reflux
CPAP: continuous positive airway pressure
OG: oro/nasogastric
FRC: functional residual capacity
OHSU: Oregon Health & Science University
NICU: neonatal intensive care unit
eCPAP: extended CPAP
dCPAP: discontinued CPAP
RCT: randomized controlled trial
SpO_2_: pulse oximeter oxygen saturation

## References

[1] Di Fiore JM, Dylag AM, Honomichl RD, Early inspired oxygen and intermittent hypoxemic events in extremely premature infants are associated with asthma medication use at 2 years of age. J Perinatol Off J Calif Perinat Assoc. 2019;39(2):203–211. doi:10.1038/s41372-018-0264-y PMC6351157 30367103

[2] Raffay TM, Dylag AM, Sattar A, Neonatal intermittent hypoxemia events are associated with diagnosis of bronchopulmonary dysplasia at 36 weeks postmenstrual age. Pediatr Res. 2019;85(3):318–323. doi:10.1038/s41390-018-0253-z PMC6377834 30538265

[3] Di Fiore JM, Bloom JN, Orge F, A Higher Incidence of Intermittent Hypoxemic Episodes Is Associated with Severe Retinopathy of Prematurity. J Pediatr. 2010;157(1):69–73. doi:10.1016/j.jpeds.2010.01.046 PMC4428609 20304417

[4] Kuzma-O’Reilly B, Duenas ML, Greecher C, Evaluation, development, and implementation of potentially better practices in neonatal intensive care nutrition. Pediatrics. 2003;111(4 Pt 2):e461–470. 12671166

[5] Ortigoza EB. Feeding intolerance. Early Hum Dev. 2022;171:105601. doi:10.1016/j.earlhumdev.2022.105601 PMC9995226 35728504

[6] Slocum C, Arko M, Di Fiore J, Martin R, Hibbs A. Apnea, bradycardia and desaturation in preterm infants before and after feeding. J Perinatol Off J Calif Perinat Assoc. 2009;29(3):209–212. doi:10.1038/jp.2008.226 PMC2745612 19148108

[7] Joshi R, Pul C van, Atallah L, Feijs L, Huffel SV, Andriessen P. Pattern discovery in critical alarms originating from neonates under intensive care. Physiol Meas. 2016;37(4):564. doi:10.1088/0967-3334/37/4/564 27027383

[8] Ambalavanan N, Weese-Mayer DE, Hibbs AM, Cardiorespiratory Monitoring Data to Predict Respiratory Outcomes in Extremely Preterm Infants. Am J Respir Crit Care Med. 208(1):79–97. doi:10.1164/rccm.202210-1971OC PMC10870840 37219236

[9] Subramaniam P, Ho JJ, Davis PG. Prophylactic or very early initiation of continuous positive airway pressure (CPAP) for preterm infants. Cochrane Database Syst Rev. 2021;10(10):CD001243. doi:10.1002/14651858.CD001243.pub4 PMC8521644 34661278

[10] Gregory GA, Kitterman JA, Phibbs RH, Tooley WH, Hamilton WK. Treatment of the Idiopathic Respiratory-Distress Syndrome with Continuous Positive Airway Pressure. N Engl J Med. 1971;284(24):1333–1340. doi:10.1056/NEJM197106172842401 4930602

[11] SUPPORT Study Group of the Eunice Kennedy Shriver NICHD Neonatal Research Network, Finer NN, Carlo WA, Early CPAP versus surfactant in extremely preterm infants. N Engl J Med. 2010;362(21):1970–1979. doi:10.1056/NEJMoa0911783 PMC3071534 20472939

[12] Morley CJ, Davis PG, Doyle LW, Brion LP, Hascoet JM, Carlin JB. Nasal CPAP or Intubation at Birth for Very Preterm Infants. N Engl J Med. 2008;358(7):700–708. doi:10.1056/NEJMoa072788 18272893

[13] Mamidi RR, Go MDA, Harris J, Extended Continuous Positive Airway Pressure in Infants Born Preterm Decreases Intermittent Hypoxemia: A Secondary Analysis of a Randomized Controlled Trial. J Pediatr. 2026;296:115165. doi:10.1016/j.jpeds.2026.115165 PMC13339869 42190903

[14] McEvoy CT, MacDonald KD, Go MA, Extended continuous positive airway pressure in preterm infants increases lung growth at 6 months. Am J Respir Crit Care Med. 2025;In Press. 10.1164/rccm.202411-2169OC PMC12005030 39977011

[15] Lam R, Schilling D, Scottoline B, The Effect of Extended Continuous Positive Airway Pressure on Changes in Lung Volumes in Stable Premature Infants: A Randomized Controlled Trial. J Pediatr. 2020;217:66–72.e1. doi:10.1016/j.jpeds.2019.07.074 PMC7986570 31519441

[16] McEvoy CT, MacDonald KD, Go MA, Extended Continuous Positive Airway Pressure in Preterm Infants Increases Lung Growth at 6 Months: A Randomized Controlled Trial. Am J Respir Crit Care Med. 2025;211(4):610–618. doi:10.1164/rccm.202411-2169OC PMC12005030 39977011

[17] Mukhtar AI, Stothers JK. Cardiovascular effects of nasogastric tube feeding in the healthy preterm infant. Early Hum Dev. 1982;6(1):25–30. doi:10.1016/0378-3782(82)90054-8 6799269

[18] Blondheim O, Abbasi S, Fox WW, Bhutani VK. Effect of enteral gavage feeding rate on pulmonary functions of very low birth weight infants. J Pediatr. 1993;122(5):751–755. doi:10.1016/S0022-3476(06)80021-1 8496756

[19] Hammerman C, Kaplan M. Oxygen Saturation during and after Feeding in Healthy Term Infants. Biol Neonate. 2009;67(2):94–99. doi:10.1159/000244149 7766736

[20] Heldt GP. The Effect of Gavage Feeding on the Mechanics of the Lung, Chest Wall, and Diaphragm of Preterm Infants. Pediatr Res. 1988;24(1):55–58. doi:10.1203/00006450-198807000-00014 3137518

[21] Pitcher-Wilmott R, Shutack JG, Fox WW. Decreased lung volume after nasogastric feeding of neonates recovering from respiratory disease. J Pediatr. 1979;95(1):119–121. doi:10.1016/S0022-3476(79)80103-1 113516

[22] Kerr P, Shoenut JP, Millar T, Buckle P, Kryger MH. Nasal CPAP Reduces Gastroesophageal Reflux in Obstructive Sleep Apnea Syndrome. Chest. 1992;101(6):1539–1544. doi:10.1378/chest.101.6.1539 1600771

[23] Kerr P, Shoenut JP, Steens RD, Millar T, Micflikier AB, Kryger MH. Nasal Continuous Positive Airway Pressure: A New Treatment for Nocturnal Gastroesophageal Reflux? J Clin Gastroenterol. 1993;17(4):276. 10.1097/00004836-199312000-00002 8308210

[24] Shepherd K, Hillman D, Holloway R, Eastwood P. Mechanisms of nocturnal gastroesophageal reflux events in obstructive sleep apnea. Sleep Breath. 2011;15(3):561–570. doi:10.1007/s11325-010-0404-x 20711680

[25] Fournier MR, Kerr PD, Shoenut JP, Yaffe CS. Effect of nasal continuous positive airway pressure on esophageal function. J Otolaryngol. 1999;28(3):142–144. 10410345

[26] Djeddi D, Cantin D, Samson N, Praud JP. Nasal Continuous Positive Airway Pressure Inhibits Gastroesophageal Reflux in Newborn Lambs. PLOS ONE. 2014;9(9):e107736. doi:10.1371/journal.pone.0107736 PMC4167239 25226514

[27] Martin RJ, Wang K, Köroğlu Ö, Di Fiore J, Kc P. Intermittent Hypoxic Episodes in Preterm Infants: Do They Matter? Neonatology. 2011;100(3):303–310. doi:10.1159/000329922 PMC3252018 21986336

[28] Miller MJ, Carlo WA, Martin RJ. Continuous positive airway pressure selectively reduces obstructive apnea in preterm infants. J Pediatr. 1985;106(1):91–94. doi:10.1016/S0022-3476(85)80475-3 3917503

[29] Krouskop RW, Brown EG, Sweet AY. The early use of continuous positive airway pressure in the treatment of idiopathic respiratory distress syndrome. J Pediatr. 1975;87(2):263–267. doi:10.1016/S0022-3476(75)80599-3 1097619

[30] Richardson CP, Jung AL. Effects of Continuous Positive Airway Pressure on Pulmonary Function and Blood Gases of Infants with Respiratory Distress Syndrome. Pediatr Res. 1978;12(7):771–774. doi:10.1203/00006450-197807000-00006 358112

[31] Bamat N, Jensen EA, Kirpalani H. Duration of continuous positive airway pressure in premature infants. Semin Fetal Neonatal Med. 2016;21(3):189–195. doi:10.1016/j.siny.2016.02.005 PMC4902733 26948885

